# Rheumatic Fever and Rheumatic Heart Disease-Related Knowledge, Attitude, and Practice in Saudi Arabia

**DOI:** 10.7759/cureus.19997

**Published:** 2021-11-29

**Authors:** Abdulmalk A Almadhi, Mohammad R Alshammri, Noora O Altamimi, Shahd A Hadal, Abdulrahman A Al Madhi, Majdi S Salahie

**Affiliations:** 1 Medicine, Imam Mohammad Ibn Saud Islamic University, Riyadh, SAU; 2 Cardiology, Imam Mohammad Ibn Saud Islamic University, Riyadh, SAU

**Keywords:** attitudes, knowledge, awareness, rheumatic heart disease, rheumatic fever

## Abstract

Objectives

Rheumatic fever (RF) is an inflammatory disorder caused by group A streptococcal pharyngitis infections that can progress to rheumatic heart disease (RHD). Public awareness and knowledge of this condition are crucial for its prevention. This study aimed to assess the knowledge and attitudes regarding these disorders to identify the factors influencing the level of knowledge and to determine how to increase awareness and knowledge of rheumatic fever and rheumatic heart disease.

Methods

An observational, cross-sectional study was conducted using a self-administered questionnaire distributed to 1211 participants throughout Saudi Arabia using an online platform. The questionnaire collected data on sociodemographic characteristics, levels of awareness, knowledge of rheumatic fever along with rheumatic heart disease, and attitudes toward these diseases.

Results

A total of 1121 participants met the criteria for the study and completed the questionnaire (77.5% female vs. 22.5% male). The most common age group was 18 to 30 years old (30.5%). The lack of knowledge was most common among the younger age group (≤ 40 years) and males. Knowledge of rheumatic fever was assessed as poor, fair, and good among 80.2%, 16.2%, and 3.6% of participants, respectively. A good knowledge level was more common among the older age group (> 40 years) and those who had four to seven children. Poor, fair, and good attitude levels were expressed by 41.7%, 32.6%, and 25.8% of participants, respectively. Poor attitudes toward rheumatic fever and rheumatic heart disease were more common among those living in the Central region.

Conclusion

While the attitudes toward rheumatic fever and rheumatic heart disease seem adequate, significant deficiencies in the knowledge and awareness of these disorders were observed in the study population. Insufficient knowledge was primarily seen among young male participants.

## Introduction

Cardiovascular disease (CVD) is the leading cause of mortality worldwide, contributing to 31% of all deaths [1]. Cardiovascular disease is becoming a significant health concern in Saudi Arabia, which is estimated to account for more than 45% of all deaths [2]. Rheumatic fever (RF) is an inflammatory disorder mediated by an abnormal immune response to group A streptococcal (GAS) pharyngitis infections, which can lead to rheumatic heart disease (RHD). Rheumatic heart disease is a chronic life-threatening condition that can progress from clinically silent to severe cardiac valve damage [3]. The possibility of developing chronic RHD remains a significant public health problem, particularly in developing countries.

It is estimated that there are 282,000 new RHD cases and 233,000 associated deaths annually, with 15 million total cases worldwide. Carapetis et al. reported that 80% of the 15.6 million RHD cases are in developing nations and that Saudi Arabia is geographically located in the region with a high prevalence of RHD [4-5]. The last study on RHD in Saudi Arabia, conducted in 1991, showed the prevalence to be 0.3 per 1000, with a prevalence of 2.8 per 1000 reporting chronic RF. In comparison, the mean incidence of acute RF among school-aged children in the United States is 19 per 100,000, and 153 to 380 per 100,000 for Australian children aged between 4 and 15 years [5-6]. In New Zealand, an average of 172 new RF cases resulted in hospitalization each year from 2010 to 2014. [7]

Case-control studies have demonstrated that a low level of maternal education is associated with RF. As pharyngitis is known to initiate RF, access to primary healthcare is crucial in reducing the incidence of this disorder. In addition, since it has been established that low socioeconomic status is associated with poor access to primary healthcare, both are well-known risk factors for RF [7].

The prevention of acute RF can be primary, secondary, and primordial by improving social conditions, such as better sanitation and housing. Using antibiotics to treat streptococcal pharyngitis is the primary prevention against acute RF, and continuous antimicrobial therapy is indicated as secondary prevention to avoid a recurrence in documented patients [7-9]. 

Group A streptococcus transmission is facilitated by close human contact. Thus, public knowledge and awareness regarding a sore throat and the transmission mechanism, prevention techniques, and management are critical for controlling acute RF [10]. Therefore, spreading awareness and knowledge among the general population in Saudi Arabia with controlling modifiable risk factors will help decrease RHD incidence and increase the quality of life [11].

Most studies have found that the level of awareness of RF and RHD is modest. Unfortunately, the number of published articles regarding RF awareness is limited, especially in Saudi Arabia. However, recent evidence from Cameroon suggests that the population lacks knowledge about all aspects of RHD and its treatment [12]. An Indian study of school-aged children's awareness of RHD also found the level of awareness to be modest [13]. Moreover, a survey of housewives showed that the majority of mothers required more education regarding acute RF symptoms, complications, routes of infection, and prevention [14].

## Materials and methods

After obtaining Imam Mohammad Ibn Saud Islamic University’s Institutional Review Board (IRB) approval (approval no.: 107-2021), an observational cross-sectional study was conducted in Riyadh, Saudi Arabia. Due to COVID-19 preventive measures, it was not feasible to conduct a community-based sampling procedure. Therefore, the questionnaire was distributed to the five districts that cover all of Saudi Arabia based on the administrative division of regions in Saudi Arabia (Central, Eastern, Southern, Northern, and Western regions) through social media platforms using simple random sampling. The questionnaire was translated from English to Arabic, validated by back translation into English, and then both versions of the questionnaire were compared for grammar and meaning. The questionnaire used was formulated following an extensive review of the literature. A pilot study was performed on 50 participants to assess the validity, clarity, and length of the questionnaire. Furthermore, three consultant cardiologists checked the content validity and approved questions based on relevance and representativeness to the research question.

The study was carried out over six months starting in August 2021. The inclusion criteria were an age of at least 18 years and current residency in Saudi Arabia. Participants who worked/studied in the medical field were excluded to avoid bias. Participants who did not fully complete the questionnaire were also excluded. Written informed consent was obtained from all participants.

The required sample size was calculated using EPI (Epidemiological Information Package) INFO, version 7.2 (CDC, Georgia, USA). According to the software, the sample size needed was at least 385 participants, using a margin of error of ± 5%, a conﬁdence level of 95%, and a 50% expected frequency.

Statistical analysis

Descriptive statistics were used to summarize the characteristics of the overall group of respondents. The knowledge of RF was assessed using 10 questions where a correct answer for each question was coded as 1 and incorrect answers as 0. Negative questions were coded in reverse to avoid bias in the score. The total knowledge score was obtained by adding the 10 items and a score range from 0 to 10 was possible, with higher scores indicating a better knowledge of RF. By using 50% and 75% as cutoff points, the participants were classified as having a poor knowledge (< 50%), fair knowledge (50 to 75%), or good knowledge (> 75%) level.

Finally, attitudes toward RF were evaluated using a three-item questionnaire, where the correct answers were coded as 1 and incorrect answers as 0. The total attitude score was calculated by adding the scores from all three questions. A score range from 0 to 3 was generated and classified into the following three categories: 0 to 1 - poor attitudes, 2 - fair attitudes, and 3 - good attitudes.

Differences in the levels of knowledge and attitudes across participants' sociodemographic characteristics were evaluated using Chi-squared tests. Two-tailed analysis with a p < 0.05 was used as the cutoff for statistical significance. The results are reported as odds ratio (OR) and corresponding 95% confidence intervals (CI). All data analyses were performed using SPSS (Statistical Package for the Social Sciences), version 26 (Armonk, NY: IBM Corp, USA).

## Results

In total, 1121 participants met the inclusion criteria. Table 1 presents the sociodemographic characteristics of the participants. The most common age group was 18 to 30 years, the majority of participants were female (77.5%), and nearly 40% resided in the Central region. With respect to monthly income, 34.2% were earning 10,001 to 20,000 Saudi Riyal (SAR) per month, and 39.4% had four to seven children. University degree holders constituted most of the participants (62.9%), 38.4% were working, and 21.9% were retired.

**Table 1:** Sociodemographic characteristics of the participants (n = 1121) SAR: Saudi Riyal

Study Data	n (%)
Age group	
18–30 years	342 (30.5%)
31–40 years	217 (19.4%)
41–50 years	309 (27.6%)
51–60 years	213 (19.0%)
> 60 years	40 (3.5%)
Gender	
Male	252 (22.5%)
Female	869 (77.5%)
Residence region	
Western region	161 (14.4%)
Southern region	118 (10.5%)
Northern region	176 (15.7%)
Eastern region	220 (19.6%)
Central region	446 (39.8%)
Monthly income (SAR)	
< 5,000	368 (32.8%)
5,000–10,000	318 (28.4%)
10,001–20,000	363 (34.2%)
> 20,000	52 (4.6%)
Number of children	
None	315 (28.1%)
1–3 children	312 (27.8%)
4–7 children	441 (39.4%)
> 7 children	53 (4.7%)
Educational level	
Secondary	225 (20.0%)
Diploma	124 (11.1%)
University	705 (62.9%)
Postgraduate	67 (6.0%)
Occupational status	
Unemployed	220 (19.6%)
Student	171 (15.3%)
Employed	431 (38.4%)
Retired	245 (21.9%)
Free business	54 (4.8%)

The attitudes toward RF and its treatment are shown in Table 2. It was revealed that the most common stated cause of a sore throat was bacterial or viral infection (53.1%), the most common person suggested for the proper treatment of a sore throat was a doctor (78.6%), and the most common method stated for the treatment of a sore throat was an antibiotic prescribed by a doctor. In addition, nearly 60% reported that they would sometimes go to the doctor for any incidence of sore throat in a child, while 7% preferred not to visit and would administer painkillers instead. Of those who did not want to visit the doctor, the most common reason was “there is no need” (69.6%). The proportion of respondents who were aware of preventive medications for RF was 10.3%. In addition, about 58% of the respondents knew the correct meaning of RF and most of them (91.2%) supported awareness campaigns for RF.

**Table 2:** Awareness and attitudes about rheumatic fever (RF) and its treatment (n = 1121) * Assessment of attitude toward RF † Indicates correct answer

Statement	n (%)
Causes of sore throat	
Cold drinks	336 (30.0%)
Cold weather	81 (7.2%)
Bacterial or viral infection	596 (53.1%)
All of the above	11 (1.0%)
I don’t know	97 (8.7%)
Person who suggested the appropriate treatment of sore throat *	
Personal experience	141 (12.6%)
Friend or family	99 (8.8%)
Doctor ^†^	881 (78.6%)
Treatment method for sore throat *	
Doctor’s prescription antibiotics ^†^	617 (55.0%)
Natural herbs	130 (11.6%)
Honey	134 (12.0%)
Gargling with water and salt	240 (21.4%)
Do you think it is important to go to the doctor if your child has a sore throat, or do you just take painkillers? *	
Yes, every time ^†^	371 (33.1%)
Yes, sometimes	671 (59.9%)
No, just take painkillers	79 (7.0%)
If no, state the reason (n = 79)	
Lack of time	7 (8.9%)
There is no need	55 (69.6%)
Other	17 (21.5%)
Aware of preventive medication for RF	
Yes	115 (10.3%)
No	1006 (89.7%)
RF is an inflammatory disease that usually affects children from 5 to 15 years of age when strep throat is not treated with antibiotics for long enough and may cause heart disease.	
Yes	650 (58.0%)
No	173 (15.4%)
I don’t know	298 (26.6%)
Now that you have answered this questionnaire, do you support the creation of campaigns to raise awareness about RF?	
Yes	1022 (91.2%)
No	14 (1.2%)
Maybe	85 (7.6%)

**Figure 1:**
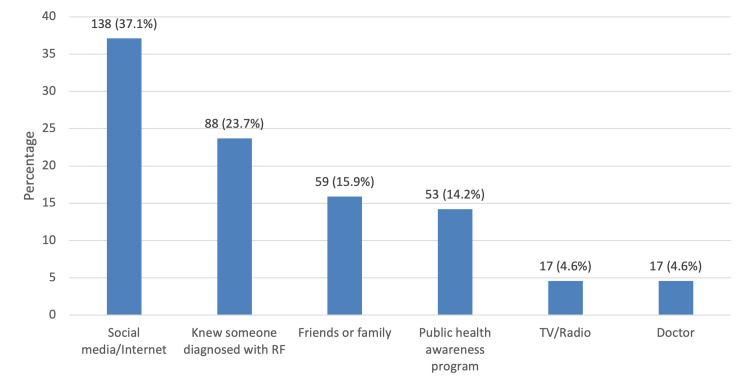
Sources for information on rheumatic fever (RF)

Figure 1 shows the sources used for information on RF. The most common source of information was social media/internet (37.1%), followed by knowing someone diagnosed with RF (23.7%) and friends or family (15.9%), while TV/radio and the doctor were the least consulted sources for information (4.6%).

The assessment of the knowledge regarding RF is shown in Table 3. It was found that 33% of the respondents knew that untreated RF can lead to heart disease. The proportion of respondents who knew that RF is not an infectious disease was 32.7%. On the other hand, 32.3% believed that improving housing and living standards is a preventive measure for RF. In addition, 29.9% of the respondents were aware that joint pain, inflammation of the heart, rash, and involuntary movement disorder are symptoms of RF. Moreover, 24.7% believed that there is a relationship between a sore throat and RF, and 20.3% knew that treating a sore throat with antibiotics prevents RF. The proportion of respondents who believed that antibiotics can be used as a preventative treatment for heart disease after RF was 16.2%. Only 12.4% knew that the age group between five to 15 years is more likely to develop RF, and only 9.7% believed that there is a relationship between bacterial dermatitis and RF. Overall, only 3.7% believed that there is no cure for heart disease caused by RF. The overall mean knowledge score was 2.15 (standard deviation [SD] 2.46), with 80.2%, 16.2%, and 3.6% of respondents classified as having a poor, fair, or good knowledge of RF, respectively.

**Table 3:** Assessment of the knowledge regarding rheumatic fever (RF). (n = 1121) † Indicates reverse answer, SD: Standard deviation

Statement	Correct n (%)
Untreated RF leads to heart disease	370 (33.0%)
RF is an infectious disease ^†^	367 (32.7%)
Improving housing and living standards is a preventive measure that helps reduce the incidence of RF	362 (32.3%)
Joint pain, inflammation of the heart, rash and involuntary movement disorders are symptoms of RF	335 (29.9%)
There is a relationship between sore throat and RF	277 (24.7%)
Treating sore throat with antibiotics prevents RF	228 (20.3%)
Antibiotics can be used as a preventative treatment for heart disease after RF	182 (16.2%)
The age group between 5 and 15 years is less likely to develop RF ^†^	139 (12.4%)
There is a relationship between bacterial dermatitis and RF	109 (9.7%)
There is a cure for heart disease caused by RF ^†^	41 (3.7%)
Total knowledge score (mean ± SD)	2.15 ± 2.46
Level of knowledge	
Poor	899 (80.2%)
Fair	182 (16.2%)
Good	40 (3.6%)

Chi-squared tests were used to determine the differences in the level of knowledge across the sociodemographic characteristics of the participants (Table 4). It was found that a prevalence of good knowledge was more common among the older age group (c2 = 13.740; p = 0.001) and those with four to seven children (c2 = 25.132; p < 0.001), while a prevalence of fair knowledge was more common among females (c2 = 18.437; p < 0.001) and those living in the Central region (c2 = 18.606; p = 0.017). Other variables, including monthly income, educational level, and occupational status, did not show a significant relationship with the level of knowledge (p > 0.05).

**Table 4:** Differences in the level of knowledge across the sociodemographic characteristics of the participants (n = 1121) § p-value was calculated using the Chi-squared test * Significant at p < 0.05

Factor	Level of knowledge			c^2^	p-value ^§^
	Poor n (%) ^(n = 899)^	Fair n (%) ^(n = 182)^	Good n (%) ^(n = 40)^		
Age group					
≤ 40 years	473 (52.6%)	71 (39.0%)	15 (37.5%)	13.740	0.001 *
> 40 years	426 (47.4%)	111 (61.0%)	25 (62.5%)		
Gender					
Male	226 (25.1%)	21 (11.5%)	5 (12.5%)	18.437	< 0.001 *
Female	673 (74.9%)	161 (88.5%)	35 (87.5%)		
Residence region					
Western region	129 (14.3%)	24 (13.2%)	8 (20.0%)	18.606	0.017 *
Southern region	91 (10.1%)	21 (11.5%)	6 (15.0%)		
Northern region	139 (15.5%)	32 (17.6%)	5 (12.5%)		
Eastern region	197 (21.9%)	18 (9.9%)	5 (12.5%)		
Central region	343 (38.2%)	87 (47.8%)	16 (40.0%)		
Monthly income (SAR)					
< 5,000	304 (33.8%)	48 (26.4%)	16 (40.0%)	8.289	0.218
5,000–10,000	256 (28.5%)	55 (30.2%)	7 (17.5%)		
11,000–20,000	295 (32.8%)	72 (39.6%)	16 (40.0%)		
> 20,000	44 (4.9%)	7 (3.8%)	1 (2.5%)		
Number of children					
None	266 (29.6%)	41 (22.5%)	8 (20.0%)	25.132	< 0.001 *
1–3 children	267 (29.7%)	37 (20.3%)	8 (20.0%)		
4–7 children	324 (36.0%)	93 (51.2%)	24 (60.0%)		
> 7 children	42 (4.7%)	11 (6.0%)	0		
Educational level					
Diploma or secondary	288 (32.0%)	52 (28.6%)	9 (22.5%)	2.289	0.318
University or postgraduate	611 (68.0%)	130 (71.4%)	31 (77.5%)		
Occupational status					
Unemployed	367 (40.8%)	78 (42.9%)	20 (50.0%)	3.692	0.449
Employed	393 (43.7%)	80 (44.0%)	12 (30.0%)		
Student	139 (15.5%)	24 (13.1%)	8 (20.0%)		

When examining differences in the level of attitudes across the sociodemographic characteristics of the participants, it was found that a good level of attitude was more common among the younger age group (c2 = 14.182; p = 0.001), while a fair level of attitude was more common among females (c2 = 6.381; p = 0.041) and those with four to seven children (c2 = 12.738; p = 0.047). On the contrary, a poor level of attitude was more common among those living in the Central region (c2 = 52.704; p < 0.001). The rest of the variables did not differ significantly across groups, including monthly income, educational level, and occupational status (p > 0.05) as seen in Table 5.

**Table 5:** Differences in the level of attitudes and practices across the sociodemographic characteristics of the participants (n = 1121) § p-value was calculated using the Chi-squared test * Significant at p < 0.05

Factor	Level of attitude & practice			c^2^	p-value ^§^
	Poor n (%) ^(n = 467)^	Fair n (%) ^(n = 365)^	Good n (%) ^(n = 289)^		
Age group					
≤ 40 years	206 (44.1%)	185 (50.7%)	168 (58.1%)	14.182	0.001 *
> 40 years	261 (55.9%)	180 (49.3%)	121 (41.9%)		
Gender					
Male	100 (21.4%)	72 (19.7%)	80 (27.7%)	6.381	0.041 *
Female	367 (78.6%)	293 (80.3%)	209 (72.3%)		
Residence region					
Western region	85 (18.2%)	53 (14.5%)	23 (08.0%)	52.704	< 0.001 *
Southern region	51 (10.9%)	41 (11.3%)	26 (09.0%)		
Northern region	41 (08.8%)	61 (16.7%)	74 (25.6%)		
Eastern region	89 (19.1%)	64 (17.5%)	67 (23.2%)		
Central region	201 (43.0%)	146 (40.0%)	99 (34.2%)		
Monthly income (SAR)					
< 5,000	146 (31.3%)	115 (31.5%)	107 (37.1%)	7.533	0.274
5,000–10,000	136 (29.1%)	103 (28.2%)	79 (27.3%)		
11,000–20,000	164 (35.1%)	134 (36.7%)	85 (29.4%)		
> 20,000	21 (4.5%)	13 (3.6%)	18 (6.2%)		
Number of children					
None	122 (26.1%)	100 (27.4%)	93 (32.2%)	12.738	0.047 *
1–3 children	126 (27.0%)	99 (27.2%)	87 (30.1%)		
4–7 children	190 (40.7%)	156 (42.7%)	95 (32.9%)		
> 7 children	29 (6.2%)	10 (2.7%)	14 (4.8%)		
Educational level					
Diploma or secondary	150 (32.1%)	103 (28.2%)	96 (33.2%)	2.244	0.326
University or postgraduate	317 (67.9%)	262 (71.8%)	193 (66.8%)		
Occupational status					
Unemployed	213 (45.6%)	136 (37.3%)	116 (40.1%)	6.530	0.163
Employed	189 (40.5%)	171 (46.8%)	125 (43.3%)		
Student	65 (13.9%)	58 (15.9%)	48 (16.6%)		

## Discussion

Deficiencies in the awareness of RF and RHD continue to be the leading adversary against better outcomes for the prevention and control of the disease. Several factors play vital roles in the level of awareness including prejudices, social and cultural beliefs, low educational levels, and poor environmental conditions. As awareness and knowledge among the public are crucial for controlling RF and RHD, this study was carried out to determine the knowledge, attitudes, and practices regarding this disease. The knowledge of the study population regarding RF and RHD was suboptimal. These results are consistent with a study carried out in Cameroon [12] where, among 256 adults and children, the level of awareness of RHD was very low, with only 5% of the respondents having adequate knowledge about the different aspects of the disease. Similar results also have been reported for a study conducted in Northern Ethiopia [15]. On the other hand, a study in Tanzania [16] reported that the overall level of awareness of RF/RHD was good and showed better outcomes than the current results.

Knowledge regarding RF and RHD is vital for an effective control program. However, in this study, the knowledge demonstrated by the participants was relatively poor. Only 3.6% had good knowledge, the majority (80.2%) had poor knowledge, and 16.2% had a fair knowledge of RF and RHD. These findings are consistent with the results of Regmi et al. [17] who reported poor knowledge about acute RF and RHD among their study population. These authors further emphasized that awareness-raising interventions are necessary to increase the knowledge about RF and RHD and to produce positive impacts on the primary interventions for these diseases. The current results also showed that age, sex, residence region, and the number of children were the relevant factors associated with knowledge of this condition. In particular, the older age group (> 40 years) and respondents with four to seven children demonstrated better knowledge, while females and those living in the Central region showed more fair knowledge. Kamal et al. [6] have also reported similar outcomes, with knowledge of RF significantly associated with age, gender, and occupation. Nkoke et al. [12] also noted that an age ≤ 35 years, a post-secondary level of education, and having heard of RHD were significantly associated with a fair knowledge of RHD.

In the current study, further assessments showed that the gaps in knowledge stemmed from various factors including a poor knowledge regarding untreated RF (33%), that RF is an infectious disease (32.7%), influential factors reducing RF (32.3%), symptoms of RF (29.9%), the relationship between a sore throat and RF (24.7%), treatments to prevent RF (16.2%), and the appropriate medications for the prevention of RF (16.2%). Likewise, knowledge was almost non-existent for the following indicators: “There is no cure for heart disease caused by RF” (3.7%), “There is a relationship between bacterial dermatitis and RF” (9.7%), and “The age group between 5 and 15 years are more likely to develop RF” (12.4%). Collectively, these results indicate that greater efforts are needed to educate the public on this disease and to provide more information on the aforementioned relevant factors.

With regard to attitudes and practices on RF, we noted that the level of the participants’ attitudes was generally better than their knowledge. Nearly 60% of the study population demonstrated a positive attitude (fair: 32.6% and good: 25.8%), while 41.7% expressed a negative attitude. It was also observed that the younger age group (≤ 40 years) exhibited better attitudes than the older group, while a fair attitude was more common among females and those with four to seven children. These findings are in accordance with the results of Kamal et al. [6], who reported a good attitude regarding RF in the general population that was associated with age, gender, and accommodation (p < 0.05).

Other factors also contributed to the decreased awareness of the study participants. For instance, few participants (10.3%) were aware of the preventive medication for RF. On the other hand, more than half (53.1%) knew that the most common cause of a sore throat was a bacterial or viral infection, most of them (78.6%) preferred a doctor to prescribe medication, and 55% reported that prescribed antibiotics were the best treatment method for a sore throat. These findings are comparable to the results of Baker et al. [7], which indicated that almost all of their participants had heard about RF, that most would go to the doctor or nurse straight away for any symptoms, and were aware that antibiotics are the most common treatment for RF. On the other hand, the study noted that the most commonly stated causes of RF were soreness of the throat and cold weather. Poor awareness of RHD also has been noted by Nkoke et al. [12], where most of their participants did not know about RHD (82%), more than 70% did not know that a sore throat can be associated with heart disease, 73% did not know what causes a sore throat, and 71% were unaware of any complications that could arise from a poorly treated sore throat. Conversely, it is interesting to note that most people in rural areas of Northern Ethiopia would take children with a sore throat to traditional healers rather than conventional healthcare providers [15]. This latter observation may be due, in part, to a lack of access to medical care in these areas, and thus an increased tendency to avail of alternative options.

Obtaining information from various sources is vital to increasing knowledge about RF and RHD. In this study, the most common source of RF and RHD information was social media/internet, followed by knowing someone diagnosed with RF and friends or family members. However, in Nepal, it was reported that the most common sources of information for these diseases were teachers, healthcare workers, and the radio [17]. It was assumed that teachers were the main source of information in this latter study since, unlike the current study, the participants were care seekers attending lectures about awareness-raising interventions for throat infections, acute RF, and RHD.

The present study has several limitations. In particular, this study used an online survey that allowed only those who had internet access to participate. Thus, some segments of the population (e.g., the elderly) may have been unintentionally missed due to poor internet accessibility.

## Conclusions

While the attitudes and practices toward RF and RHD seem adequate, deficiencies in the knowledge and awareness of these conditions were observed in the study population, primarily among young male adults. Thus, there is a need to raise knowledge and awareness of RF and RHD among the study population to help improve the prevention and control of RF and RHD in our region.
